# Proteomic profiling of murine biliary-derived hepatic organoids and their capacity for drug disposition, bioactivation and detoxification

**DOI:** 10.1007/s00204-021-03075-3

**Published:** 2021-05-29

**Authors:** Lawrence Howell, Rosalind E. Jenkins, Stephen Lynch, Carrie Duckworth, B. Kevin Park, Christopher Goldring

**Affiliations:** Department of Pharmacology and Therapeutics, MRC Centre of Drug Safety Science, University of Liverpool, The Sherrington Building, Ashton Street, Liverpool, L69 3GE UK

**Keywords:** In vitro, Organoid, Drug-metabolizing enzymes and transporters, Hepatic phenotype

## Abstract

Hepatic organoids are a recent innovation in in vitro modeling. Initial studies suggest that organoids better recapitulate the liver phenotype in vitro compared to pre-existing proliferative cell models. However, their potential for drug metabolism and detoxification remains poorly characterized, and their global proteome has yet to be compared to their tissue of origin. This analysis is urgently needed to determine what gain-of-function this new model may represent for modeling the physiological and toxicological response of the liver to xenobiotics. Global proteomic profiling of undifferentiated and differentiated hepatic murine organoids and donor-matched livers was, therefore, performed to assess both their similarity to liver tissue, and the expression of drug-metabolizing enzymes and transporters. This analysis quantified 4405 proteins across all sample types. Data are available via ProteomeXchange (PXD017986). Differentiation of organoids significantly increased the expression of multiple cytochrome P450, phase II enzymes, liver biomarkers and hepatic transporters. While the final phenotype of differentiated organoids is distinct from liver tissue, the organoids contain multiple drug metabolizing and transporter proteins necessary for liver function and drug metabolism, such as cytochrome P450 3A, glutathione-*S*-transferase alpha and multidrug resistance protein 1A. Indeed, the differentiated organoids were shown to exhibit increased sensitivity to midazolam (10–1000 µM) and irinotecan (1–100 µM), when compared to the undifferentiated organoids. The predicted reduced activity of HNF4A and a resulting dysregulation of RNA polymerase II may explain the partial differentiation of the organoids. Although further experimentation, optimization and characterization is needed relative to pre-existing models to fully contextualize their use as an in vitro model of drug-induced liver injury, hepatic organoids represent an attractive novel model of the response of the liver to xenobiotics. The current study also highlights the utility of global proteomic analyses for rapid and accurate evaluation of organoid-based test systems.

**Supplementary Information:**

The online version contains supplementary material available at 10.1007/s00204-021-03075-3.

## Introduction

Drug-induced liver injury (DILI) results in significant financial costs to the pharmaceutical industry. Therefore, a predictive and proactive approach in determining DILI as a potential outcome during drug development is warranted. While necessary, current in vitro models of hepatotoxicity are insufficiently predictive of the mechanisms and severity of DILI, for both pre-clinical animal models and humans (Weaver et al. 2020). Primary hepatocytes have often been described as the gold-standard cell model for hepatotoxicity testing. However, their availability, stability during long-term culture and high inter-individual variation limit their utility. Furthermore, their inability to proliferate makes them an expensive single-use option. Therefore, the use of proliferative cell lines is commonplace. A portfolio of cell lines of varying complexity, such as HepG2, HepaRG and Upcyte cells, cultured in 2D or 3D, signify a varied representation of the in vivo phenotype (Sison-Young et al. 2015). However, to date, there is no single cell culture model that sufficiently satisfies the wide-reaching requisites of DILI prediction (Gerets et al. 2012; Hughes et al. 2007; Rogue et al. 2012).

The liver is responsible for many key physiological processes in the body, which is reflected in its abundant and diverse proteome. Nevertheless, current in vitro models poorly represent the in vivo organ phenotype. For example, a common criticism of the existing in vitro models is the low-level expression or gradual depletion of key drug-metabolizing enzyme and transporter (DMET) proteins, which is essential when evaluating potential hepatotoxicity of compounds (Choi et al. 2015; Heslop et al. 2017). Furthermore, key cellular defense proteins and pathways involved in the response to a toxic insult, such as the nuclear factor erythroid 2-related factor 2/Kelch-like ECH-associated protein 1 (NRF2-KEAP1) signaling cascade, may also be dysregulated. This may be an inherent attribute due to the source of the model (e.g., cancer-derived cell lines) or as an adapted response over time to cell culture conditions (Ishii and Mann 2014; Shibata et al. 2008). Poorly predictive in vitro models ultimately necessitate the use of multiple, expensive and ethically challenging pre-clinical models to calculate hepatotoxicity, which in themselves are not absolutely predictive (Olson et al. 2000). Therefore, there is an unmet need for an in vitro hepatic model that shows physiological improvement over the currently used systems.

Organoids are a recent innovation in in vitro modeling. They are described as a 3D cell model consisting of self-organizing, organ-specific cell types that mimics the corresponding in vivo tissue (Method of the year 2017: Organoids 2018). They are typically derived from pluripotent or resident adult stem cells within a primary tissue. Organoid development is generally driven through specific cell culture conditions, such as extracellular matrix formation and growth factor-driven differentiation. Organoids have garnered interest as an attractive physiological model due to their ability to recapitulate the phenotype of their donor tissue in both rodents and humans. For example, α1-antitrypsin deficiency and Alagille syndrome have both been modeled using patient-derived hepatic organoids (Huch et al. 2015), and organoids derived from pancreatic cancer formed carcinomas when orthotopically transplanted into mice (Boj Sylvia et al. 2015).

To date, the primary reported use of organoids has been to model physiological disease states (Xu et al. 2018). However, the use of hepatic organoids as a model of DILI remains to be explored. It has been demonstrated that biliary-derived organoids are capable of cytochrome P450 3A4 (CYP3A4) mediated detoxification of midazolam, although the metabolism of additional drugs by different CYP450 families remains to be elucidated. As hepatic organoids are reported to maintain genetic stability throughout long-term culture and produce a mature hepatocyte-like phenotype when fully differentiated, they may offer an improvement over currently available hepatotoxicity models (Huch et al. 2015).

While hepatic organoids could be considered a nascent model of DILI, they are yet to be fully characterized to a comparable standard to pre-existing models. The assessment of the suitability of hepatic organoids as a model for hepatotoxicity is dependent on DMET and NRF2 protein expression when compared to liver. Global proteomic profiling of hepatic organoids and the livers from which they were derived would allow a direct comparison of their relative phenotypes. In this study, therefore, the proteomes of undifferentiated and differentiated hepatic organoids and donor-matched liver tissue were compared by mass spectrometry to assess phenotypic differences. From a toxicological perspective, we hypothesized that differentiation would induce key CYP450, phase II and transporter proteins to a phenotype comparable to liver tissue, and position hepatic organoids as a potential in vitro model for pre-clinical drug and hepatotoxicity testing.

## Materials and methods

Unless otherwise stated, all organoid reagents were purchased from STEMCELL technologies (Grenoble, France) and general reagents were purchased from Sigma Aldrich (Poole, UK).

### Experimental animals

The protocols described were undertaken in accordance with the criteria outlined in a project license granted under the Animals Scientific Procedures Act 1986 and approved by the University of Liverpool Animals Ethics Committee. 5- to 7-week-old male CD-1 mice were purchased from Charles River laboratories (Cambridge, UK) and had a 7-day acclimatization period prior to use. Animals were maintained in a 12 h light/dark cycle with free access to food and water.

### Isolation of murine hepatic duct fragments

Male 6- to 8-week-old CD-1 mice were culled by schedule 1 cervical dislocation. The liver was removed, dissected into 3–5 mm pieces and incubated with digestion solution [Dulbecco’s Modified Eagle’s Medium (DMEM)/F12 supplemented with 15 mM 4-(2-hydroxyethyl)-1-piperazineethanesulfonic acid (HEPES), collagenase IV (1 mg/mL) and dispase (1 U/mL)] at 37 °C. Fresh digestion solution was added every 20 min and the previous supernatant was removed and retained on ice, until the liver was fully digested. The pooled digestion mixture was passed through a 70 µm filter and then through a reversible 37 µm strainer and the cell suspension discarded. The filter was inverted, and ductal fragments captured on the strainer were eluted into ice-cold DMEM/F12 media. The fragments were then pelleted at 300 × *g* at 4 °C for 5 min.

### Culturing and differentiation of hepatic organoids

Growth factor-reduced Matrigel (356231, Corning, MA, USA) was added to the pelleted ductal fragments and gently resuspended by pipette. Forty microliter of the mixture was pipetted into the middle of well of a pre-warmed 24-well plate. The plate was incubated at 37 °C for 15 min to allow the Matrigel suspension to solidify into a dome. Aliquots of 700 µL of HepatiCult media were added per well and the organoids were incubated at 37 °C in a humidified atmosphere of 5% CO_2_. Organoids were monitored daily, with media changes every 2–3 days.

At the start of differentiation, organoids were passaged and grown in HepatiCult media to promote outgrowth from fragments into organoids for 3 days. The medium was changed to differentiation medium (Advanced DMEM/F12 supplemented with 1% penicillin/streptomycin, 1% GlutaMAX, 10 mM HEPES, 1:50 B27 supplement with vitamin A, 1 mM N-acetylcysteine, 10 μM DAPT, 10 nM recombinant human [Leu15]-gastrin I, 50 ng/mL recombinant mouse epidermal growth factor (Peprotech, US), 100 ng/mL recombinant human fibroblast growth factor 10 (Peprotech) and 50 nM A83-01 (Tocris Bioscience, Bristol, UK)) as defined by Broutier et al*.* (2016) for 9 days. To promote complete differentiation, the media was supplemented with 3 µM dexamethasone for 3 days. The media was changed daily throughout the entire differentiation protocol.

### Immunofluorescence analysis of hepatic markers

Organoids were fixed with 2% paraformaldehyde for 20 min and washed three times with phosphate buffered saline (PBS) with 0.1 M glycine. Organoids were permeabilized with PBS containing 0.5% Triton-X for 10 min and washed three times with immunofluorescence wash buffer (PBS with 0.25% Triton-X and 0.05% Tween-20) for 10 min.

Organoids were then incubated for 1 h in blocking buffer (immunofluorescence wash buffer with 10% casein blocking solution). The blocking solution was removed and replaced with the primary antibodies diluted in blocking solution overnight at 4 °C. The following antibodies were used: cytokeratin 19 (CK19) [ab52625, Abcam (Cambridge, UK), 1:200], alpha-fetoprotein (AFP) (ab213328, Abcam, 1:100), albumin (ab207327, Abcam, 1:500) and CYP2E1 (ab28146, Abcam, 1:1000).

The primary antibody solution was removed, the organoids were then washed in blocking solution, and then incubated with goat anti-rabbit IgG Alexa Fluor 488 (ab150077, Abcam, 1:500) and phalloidin 594 (ab176757, Abcam, 1:500) in blocking buffer for 1 h. The organoids were then incubated with PBS containing Hoechst (1:10,000) for 10 min. 100 µL of PBS was added to the well to prevent Matrigel domes from drying and organoids were imaged on an Axio observer Z1 microscope (Zeiss, Germany).

### Isobaric tagging for relative and absolute quantitation (iTRAQ) and LC–MS/MS analysis

Organoids were broken from their Matrigel domes and washed three times in ice-cold PBS and pelleted at 300 × *g* at 4 °C for 5 min. Undifferentiated organoids, fully differentiated hepatic organoids and donor-matched liver tissue (*n* = 4 per group) were prepared for iTRAQ analysis. Liver samples were homogenized, and all samples were mixed with dissolution buffer (0.5 M triethylammonium bicarbonate, 0.1% SDS, pH 8.5) and processed with a sonicator (3 × 10 s, 5 µm amplitude). The samples were centrifuged at 14,000 × *g* at 4 °C for 15 min, and the samples were diluted in dissolution buffer to a protein concentration of 5 mg/mL. A pool containing equal quantities of all the samples was prepared and included in duplicate in each of the two iTRAQ runs.

For each sample, 100 µg of protein was denatured, reduced, cysteine blocked, digested and labeled by iTRAQ Reagents Multiplex Kit (Sciex, USA) according to the manufacturer's protocol. Unbound reagent was removed by cation exchange chromatography using a Polysulfoethyl A column and fractions were desalted using a macroporous C18 column (Agilent, USA) and dried by centrifugation under vacuum (SpeedVac, Eppendorf, Germany). Samples were analyzed by LC–MS/MS on a Triple TOF 6600 mass spectrometer (Sciex), delivered into the instrument by automated in-line liquid chromatography using an Eksigent nanoLC 400 System mounted with a NanoAcquity 5 µm, 180 µm × 20 mm C_18_ trap and 1.7 µm, 75 µm × 250 mm analytical column (Waters, USA). Spectra were acquired in positive ion mode with up to 25 MS/MS spectra acquired per 2.5 s cycle throughout the course of the 90 min LC gradient. 

### iTRAQ protein identification and statistical analyses

Protein identification and relative quantification were performed using ProteinPilot 5 (Sciex) and the SwissProt database (July 2018, 16,985 mouse proteins). Proteins identified with more than 95% confidence and within a global FDR of 1% (decoy reversed database) were included in the statistical analysis. Ratios for each iTRAQ label were obtained using the common pools as the denominator. Data from the two iTRAQ runs were merged using RStudio V.1.0.143. Ratios were converted to their natural log and data analysis was performed using Partek Genomic Suite software V.7.18.0518, (Partek, MO, US). Hierarchical cluster analysis and PCA were performed on data batch corrected for iTRAQ experiment. Proteins that were differentially expressed between the sample types were determined using a 2-way ANOVA on the uncorrected data, with iTRAQ experiment and tissue type as factors. Relevant volcano plots were derived from these data. The mass spectrometry proteomics data have been deposited to the ProteomeXchange Consortium via the PRIDE (Perez-Riverol et al. 2019) partner repository with the dataset identifier PXD017986.

### Functional annotation clustering analysis

Multiple testing corrections were employed using the Benjamini–Hochberg procedure with a false discovery rate of 10%. The corrected data were subjected to Functional Annotation Clustering using the **D**atabase for **A**nnotation, **V**isualization and **I**ntegrated **D**iscovery (DAVID, v6.8), with the full list of proteins identified in the combined iTRAQ data as the background list (Huang et al. 2009a, b). The analysis was performed with default settings (medium stringency, EASE threshold = 1) and the most significant up and down-regulated clusters were reported.

### Ingenuity pathway analysis (IPA)

Fold changes and *p* values for each of the pairwise comparisons (undifferentiated organoids v liver, differentiated organoids v liver and undifferentiated v differentiated organoids) were uploaded to Ingenuity Pathway Analysis (IPA, QIAGEN Inc., https://www.qiagenbioinformatics.com/products/ingenuity-pathway-analysis). A core analysis was performed with the Ingenuity Knowledge Base as the reference set, and with a cut-off from − 5 to 5 and a maximum *p* value of 0.05. This resulted in 1104 proteins being carried forward for analysis.

### Immunoblotting of protein lysates

20 µg of protein lysate (*n* = 2 per group) was separated by sodium dodecyl sulfate polyacrylamide gel electrophoresis. Resolved proteins were transferred using a Trans-Blot turbo transfer system (Bio-Rad, UK). Membranes were then probed with anti-alanine amino transferase 1 (ALT1) (ab202083, Abcam, 1:2000 in 2.5% milk), anti-CYP3a4 (ab3572, Abcam, 1:2000 in 2.5% milk), anti-glutathione-*S*-transferase A1 (GSTA1) (ab135709, Abcam, 1:250 in 2.5% milk), anti-peroxiredoxin 1 (ab15571, Abcam, 1:1000 in 2.5% milk) and anti-glyceraldehyde-3-phosphate dehydrogenase (GAPDH) (G9545, Sigma, 1:5000 in 2.5% milk). Proteins were visualized with a Chemidoc imaging system and band density was calculated with Image Lab software V.6.0.1 (Bio-Rad). Densitometry analysis was performed by normalizing protein expression to GAPDH and quantifying each sample relative to the pooled sample.

### Investigation of the effect of differentiation on the response of the differentiated organoids to dose-dependent toxicity

Differentiated and undifferentiated organoids were dosed with 0.1, 1 and 10 mM acetaminophen (APAP), 1,10,100 and 1000 µM midazolam and 1,10,100 µM irinotecan. Each compound was diluted to a final working concentration of 0.01% dimethyl sulfoxide (DMSO). Cell viability was measured via LDH release in the supernatant. The LDH cytotoxicity assay was performed as per the manufacturer’s instructions (Merck-Sigma). Paired *t* test analysis was performed against differentiated/undifferentiated at the equivalent dose and time to assess significance. Each well consisted of ~ 20 organoids, and each data point was completed in triplicate, *n* = 3.

## Results

### Differentiated hepatic organoids express hepatic markers of varying maturity

**Fig. 1:**
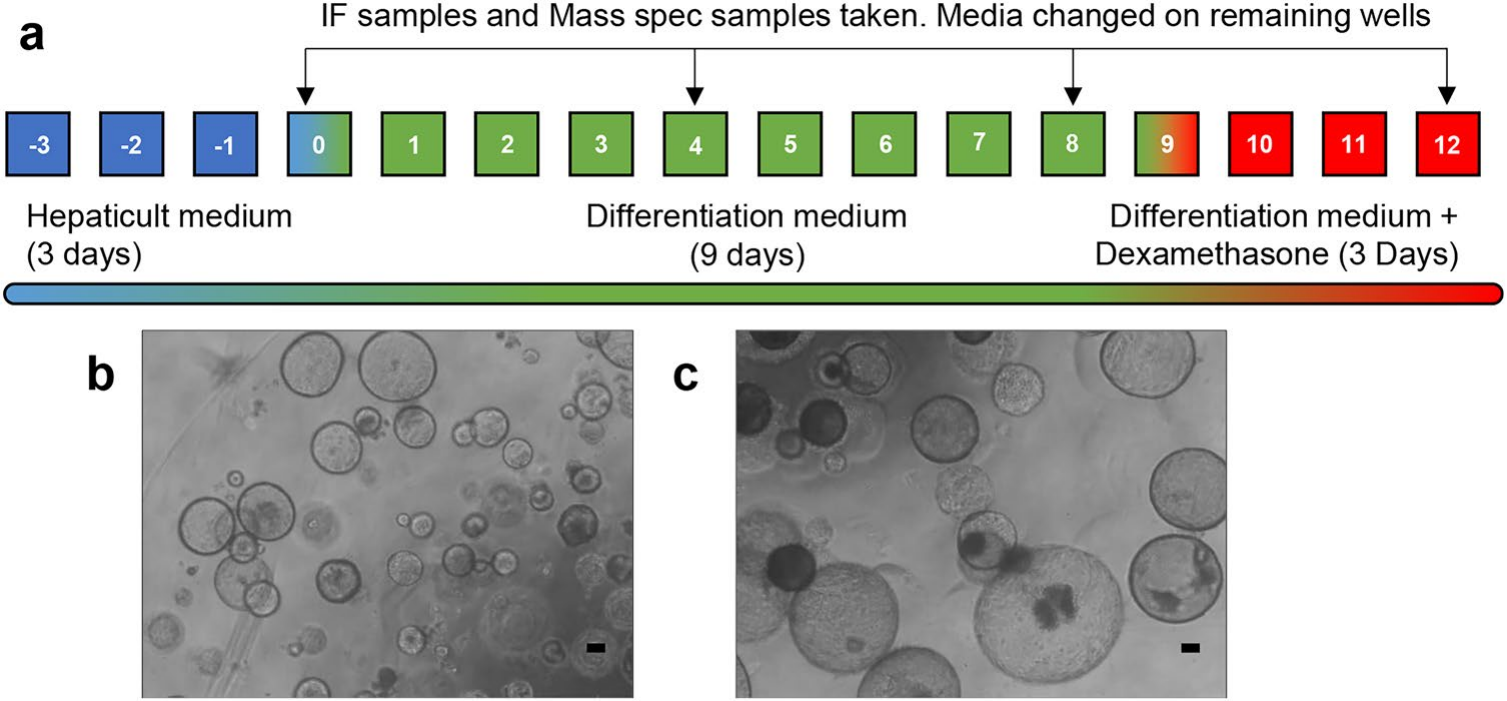
Hepatic organoids are differentiated over 15 days with defined culture media. **a** Differentiation and sample analysis timeline for hepatic organoids. Differentiation media inhibits Notch signaling, which changed organoids from a biliary phenotype to a mature hepatic phenotype. **b** Small, undifferentiated organoids were maintained in HepatiCult media, prior to differentiation. **c** Fully differentiated hepatic organoids were much larger and expressed mature hepatic markers. Scale bar 100 µm and × 20 magnification

To accurately mimic the phenotype of an in vivo liver, hepatic organoids should be able to demonstrate essential physiological attributes, such as albumin production and CYP450 expression. As organoids originate from ductal fragments, they require differentiation to exhibit a mature hepatocyte phenotype. Differentiation media contain small molecules such as A83-01 and DAPT, which promote differentiation and inhibit or interfere with Notch signaling, an essential pathway in biliary development (Lorent et al. 2004). Organoids were cultured in differentiation media for 9 days, and a further 3 days in differentiation media supplemented with dexamethasone, a known CYP450 inducer (Lu and Li 2001) (Fig. 1).

To assess the extent and suitability of the differentiation from a biliary phenotype to a mature hepatocyte phenotype, organoids were stained for actin, CYP2E1, biliary marker CK19, immature hepatocyte marker AFP and mature hepatocyte marker albumin (Supplementary Figure 1). Undifferentiated organoids had a relatively small diameter of approximately 80–250 µm and expressed both CK19 and CYP2E1. Throughout the differentiation, there was a gradual loss of CYP2E1 expression and gain of hepatocyte markers AFP and albumin by day 8. The levels of CK19 appeared to remain constant. At the completion of the differentiation protocol on day 12, organoids were significantly larger, with an approximate diameter of 300–500 µm. Organoids maintained the expression of CK19. However, expression of immature and mature hepatocyte markers was highest at this stage of the organoid culture, although this was not uniform across all organoids (Supplementary Figure 1). Notably, after the inclusion of dexamethasone, there appeared to be a slight re-emergence of CYP2E1 expression (Supplementary Figure 1, day 12).

### Global protein expression in liver, and in undifferentiated and differentiated hepatic organoids

**Fig. 2:**
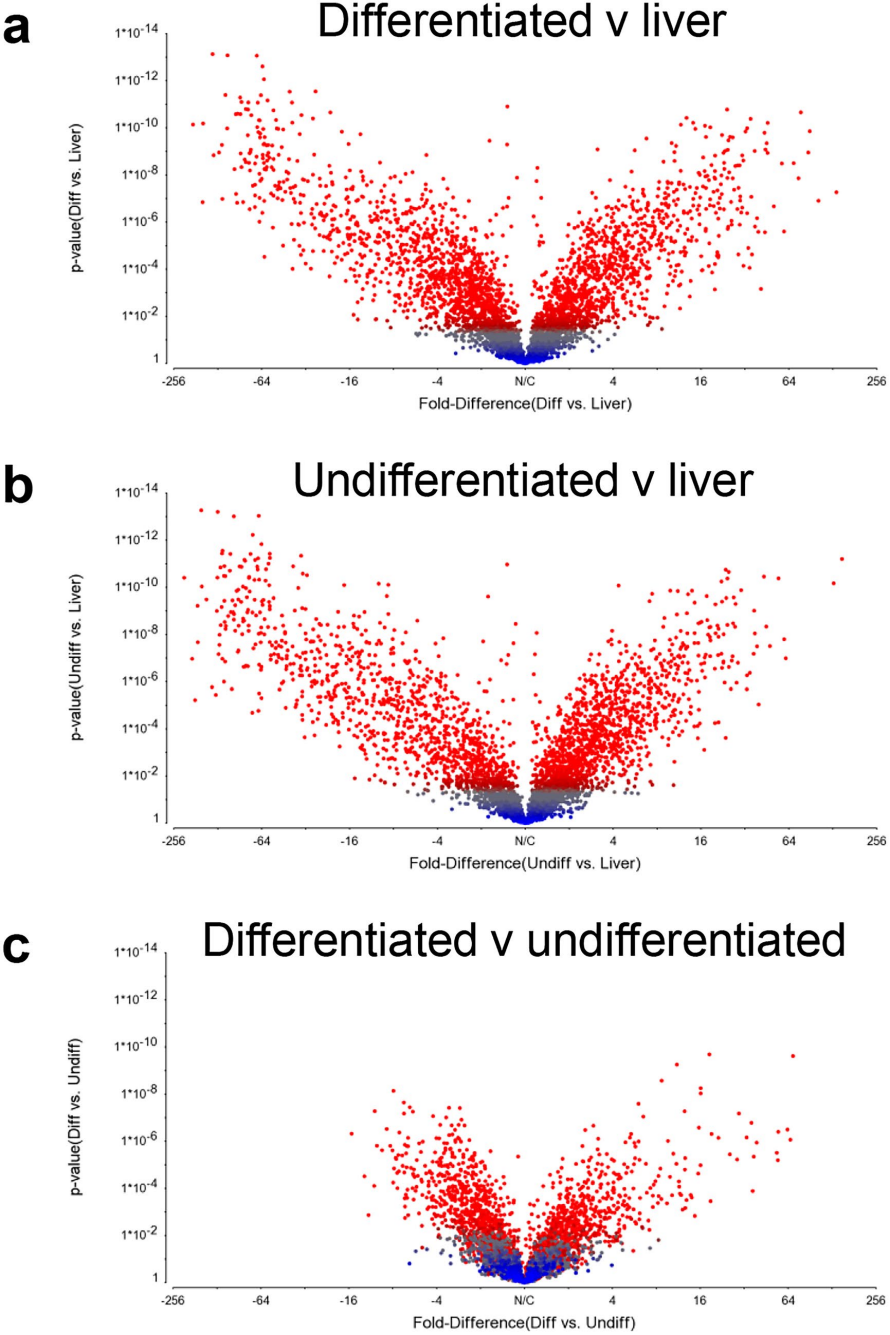
Global differential protein expression between hepatic organoids and livers varies by significance. Volcano plots of **a** differentiated organoids compared to liver, **b** undifferentiated organoids compared to liver and **c** differentiated organoids compared to undifferentiated organoids indicating statistical significance (*p* value) versus fold change. Statistical significance is defined as   = *p* < 0.05 (color figure online)

A comprehensive proteomic analysis of undifferentiated and differentiated organoids was conducted. To our knowledge, this represents the first such analysis to be undertaken, and enables clear insights to be gained into the likely functional characteristics of hepatic organoids and their ability to recapitulate pharmacologically and toxicologically relevant aspects of liver biology. To evaluate differential protein expression in liver and undifferentiated and differentiated organoids, samples were lysed and prepared for iTRAQ. LC–MS/MS identified 4405 proteins across all samples (ProteomeXchange identifier PXD017986). Principal Component Analysis (PCA) of the batch-corrected data showed distinct clustering populations of liver, differentiated and undifferentiated organoid samples (Supplementary Figure 2a). This was confirmed by hierarchical clustering, which demonstrated clustering of organoid samples when compared to liver tissue (Supplementary Figure 2b). Volcano plots comparing differentiated (Fig. 2a) and undifferentiated (Fig. 2b) organoids to liver both demonstrated a similar distribution of points, with large expression changes at a high degree of significance. Of the total 4405 proteins detected, 2725 (61.8%) and 2662 (60.4%) were significantly (*p* < 0.05) altered in differentiated and undifferentiated organoids, respectively, when compared to liver. Conversely, when differentiated and undifferentiated organoids were compared, the relative change of protein expression and level of significance was small (Fig. 2c). A total of 1740 (39.5%) proteins were significantly (*p* > 0.05) altered, further indicating both sets of organoid samples were more similar to each other than their source liver tissue.

### Expression of key hepatic proteins in liver, and in undifferentiated and differentiated hepatic organoids

**Fig. 3:**
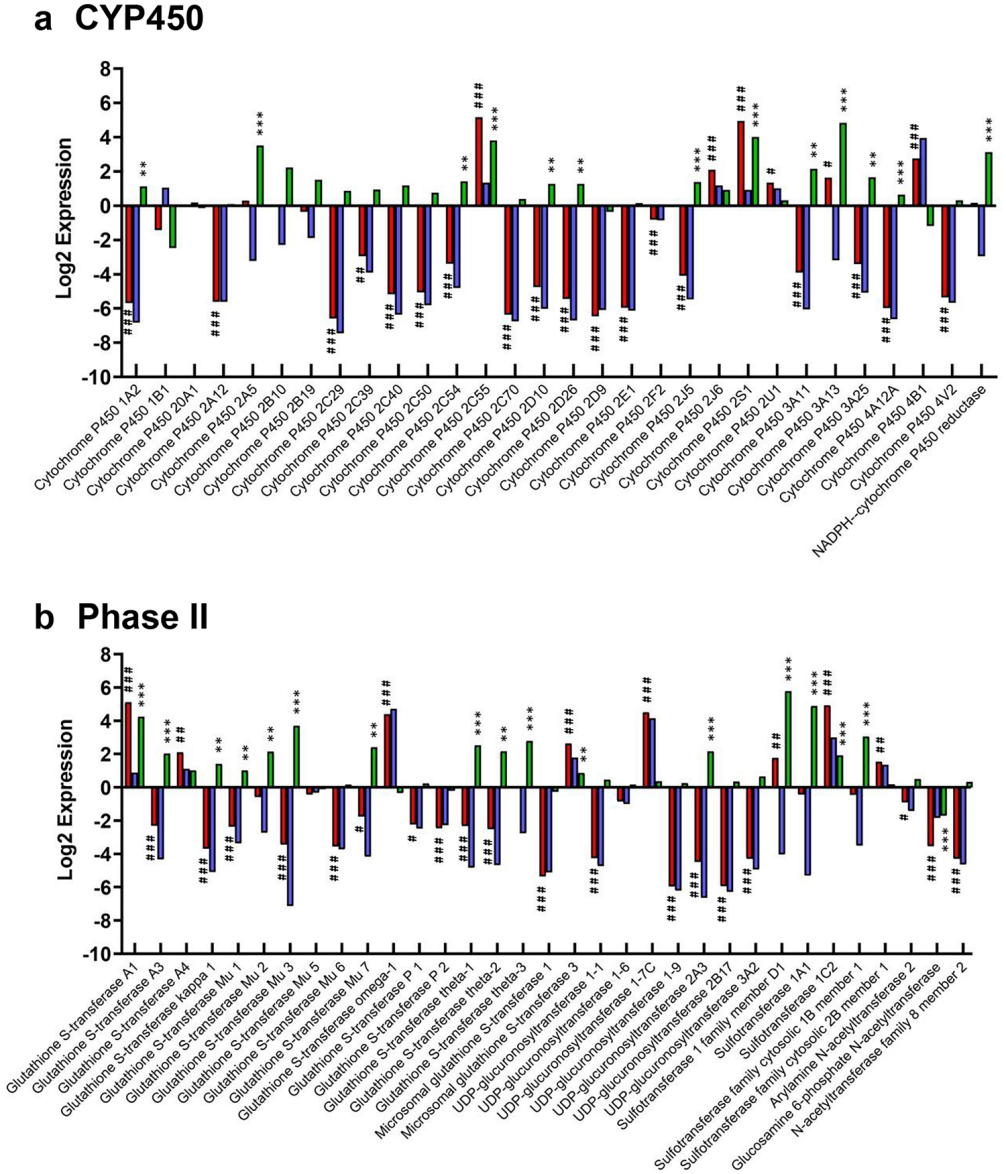

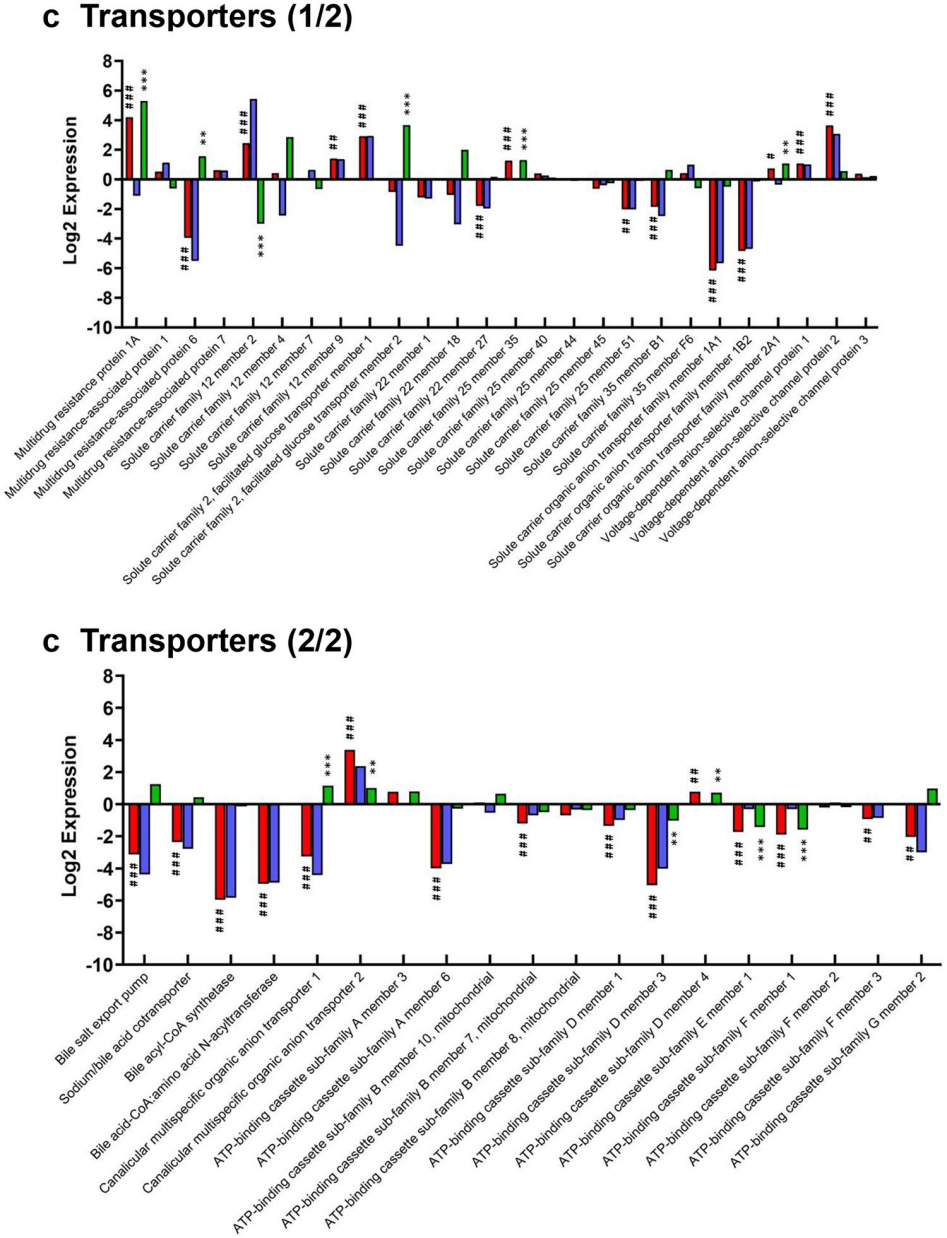

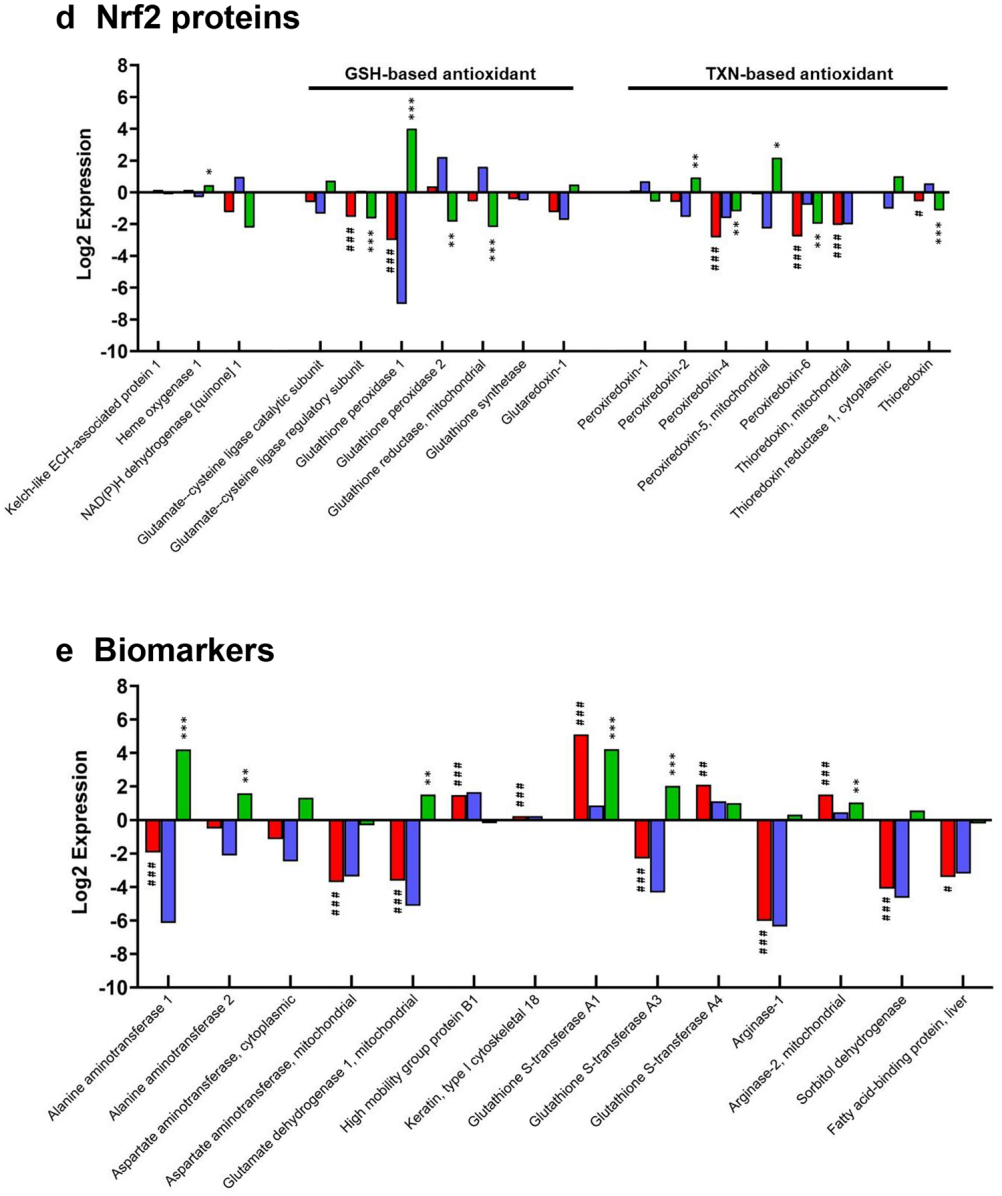
Comparison of log2 fold changes of individual key hepatic proteins in undifferentiated organoids, differentiated organoids and liver. The log2 difference in expression of **a** 30 CYP450 enzymes, **b** 30 Phase II, **c** 45 transporter, **d** 25 NRF2 and auxiliary and **e** 14 biomarker proteins was calculated following iTRAQ analysis. A hash (^#^) or asterisk (*) indicates significant differential expression of proteins in differentiated hepatic organoids versus liver or differentiated versus undifferentiated hepatic organoids, respectively. * = 0.05 > *p* > 0.01, ** = 0.01 > *p* > 0.001 and *** = *p* < 0.001.  differentiated v liver,  undifferentiated v liver,  differentiated v undifferentiated (color figure online)

Due to the implicit roles of metabolic turnover, conjugation and elimination of xenobiotics and chemicals by the liver, a focused examination of individual proteins responsible for these processes was performed. The DMET proteins of interest included 30 cytochrome P450 enzymes, 32 Phase II enzymes and 45 transporter proteins; 14 NRF2-target and 14 hepatic biomarker proteins of interest were also detected. Figure 3a shows the log2 change in expression of CYP450 enzymes between liver, differentiated and undifferentiated hepatic organoids. Levels of CYP450 in organoids were consistently much lower than in liver, except for CYP2c55, 2j6, 2s1, 2u1 and 4b1. Upon differentiation, there was a significant increase in expression of multiple CYP450s compared to the undifferentiated organoids; notable examples include CYP1a2 (2.2-fold), 2a5 (11.5-fold), 2d10 (2.4-fold), 2d26 (2.4-fold), 3a11 (4.5-fold) and 3a13 (28.6-fold). CYP3a13 was particularly sensitive to differentiation and was the only CYP450 whose abundance was significantly lower in undifferentiated hepatic organoids versus liver (9.1-fold lower) and was also significantly higher in differentiated hepatic organoids versus liver (28.6-fold higher). There was no substantial change in the expression of CYP2E1 in all three sample types, however, immunofluorescent staining demonstrated that it was expressed both pre- and post-differentiations (Supplementary Figure 1).

The expression of phase II enzymes was largely lost in hepatic organoids when compared to liver, except for various subclasses of the GST family and sulfotransferase family. Differentiation of hepatic organoids broadly reversed this trend and the majority of phase II enzymes were significantly increased. However, the *N*-acetyltransferase and UGT family was largely unchanged across all three groups, except for UDP-glucuronosyltransferase 2A3 which was 4.5-fold higher in differentiated versus undifferentiated organoids (Fig. 3b).

Transporters were generally present at lower levels in hepatic organoids compared to livers, though not to the extent of CYP450 and phase II proteins (Fig. 3c). There was no significant change in solute carrier family 22 member 1 (OCT1) or multidrug resistance-associated protein 1 and 7 (MRP1/7) expression in differentiated organoids compared to both liver and undifferentiated organoids. Conversely, solute carrier organic anion transporter 2A1 (OATP-2A1, 1.6-fold and 2.1-fold), multidrug resistance protein 1A (MDR1A, 18.2-fold and 38.9-fold) and canalicular multispecific organic anion transporter 2 (MRP3, 10.4-fold and 2-fold) were all significantly up-regulated in differentiated organoids compared to both liver and undifferentiated organoids (Fig. 3c).

The majority of hepatic influx and efflux proteins were significantly under-expressed in differentiated organoids relative to liver, including OATP-A1 (71-fold) and -1B2 (28.5-fold), ATP-binding cassette sub-family G member 2 (BCRP, 4.1-fold), and the bile salt export pump (BSEP, 8.7-fold). Differentiation did not significantly affect the expression of these proteins compared to undifferentiated organoids. MRP6 and canalicular multispecific organic anion transporter 1 (MRP2) were down-regulated in differentiated organoids compared to liver, although differentiation did significantly increase their expression (2.9-fold, and 2.2-fold, respectively) (Fig. 3c).

The changes in abundance of key NRF2-related proteins (Hayes and Dinkova-Kostova 2014) were also analyzed. The relative levels of KEAP1, the repressor protein of NRF2, and heme oxygenase-1 and NAD(P)H dehydrogenase [quinone] 1, two commonly used reporters of NRF2 activation, were similar between differentiated organoids and liver. However, there was some minor up- and down-regulation of GSH- and thioredoxin-based NRF2 antioxidant proteins. The largest observed effect of differentiation was upon glutathione peroxidase 1. NRF2-related protein abundance was diminished in undifferentiated organoids compared to the liver (128-fold), although this effect was significantly reduced post-differentiation (eightfold) (Fig. 3d).

To be considered as a predictive model of liver injury, the expression of currently used and recently identified putative protein biomarkers of DILI (Church et al. 2019) were also investigated. Differentiation of organoids increased the expression of alanine aminotransferase (ALT, 18.5-fold), aspartate aminotransferase (2.5-fold) and glutamate dehydrogenase (2.8-fold) compared to undifferentiated organoids, whereas high mobility group box 1 and cytokeratin-18 were unaffected. Among the candidate novel biomarkers of DILI, differentiation significantly increased the expression of the phase II enzymes GSTA1 (18.8-fold) and GSTA3 (4.1-fold) (shown in Fig. 3b), sorbitol dehydrogenase (1.5-fold) and arginase 2 (2.1-fold) (Fig. 3e).

### Functional annotation reveals distinctly altered biological pathways in differentiated hepatic organoids

Mass spectrometry analysis of differentiated organoids revealed 2487 and 1240 significantly (*p* < 0.05) differentially expressed proteins when compared to liver and undifferentiated organoids, respectively, after multiple testing correction using Benjamini–Hochberg (10% FDR). These changes in abundance were observed both globally (Fig. 2, uncorrected data) and in DMET- and NRF2-associated proteins (Fig. 3a–e). To understand the implications of these changes in expression, functional annotation cluster analysis (DAVID) was used to contextualize significantly up and down-regulated proteins within the different samples. The annotation clusters with the most significant enrichment scores for each condition are reported.

Proteins associated with cytoskeletal reorganization and cell–cell adhesion were up-regulated in differentiated organoids compared to liver (Table 1a). There was also an increase in a number of proteins involved in mRNA processing/transcriptional regulation. Conversely, proteins associated with energy production and metabolism were down-regulated in differentiated organoids compared to liver (Table 1b). With respect to the hepatic phenotype, there was a decrease in the abundance of proteins associated with drug metabolism.

**Table 1:** Functional annotation cluster analysis (DAVID) of iTRAQ expression data

(a) Differentiated v liver up-regulated			
Annotation cluster	Count	Enrichment score	*p* value
Actin binding	65	12.11	8.22E–13
Cell–cell adhesion	117	9.73	7.58E–13
Spliceosome	54	4.07	5.60E–10
PI3K-Akt signaling	44	4.02	2.18E–05
EF hand domain	30	3.89	1.66E–04
SAP domain	11	3.73	6.67E–05
LIM domain	18	3.53	2.76E–06
SH3 domain	34	3.17	1.59E–04
Chromosome	44	3.08	1.63E–06

(b) Differentiated v liver down-regulated			
Annotation cluster	Count	Enrichment score	*p* value
Mitochondrion	457	32.54	2.59E–43
Oxidoreductase	223	25.39	1.75E–32
Respiratory chain	45	13.48	2.74E–19
Fatty acid/lipid metabolism	107	12.32	6.16E–13
Ribosomal protein	83	12.06	8.54E–15
Peroxisome	50	6.56	1.90E–10
Endoplasmic reticulum	170	5.57	3.07E–06
Flavoprotein	48	4.84	1.13E–07
Proteasome	35	4.37	2.98E–10
Cytochrome P450	29	3.51	6.34E–05
Proteolysis	25	3.18	2.14E–07

(c) Differentiated v undifferentiated up-regulated			
Annotation cluster	Count	Enrichment score	*p* value
Oxidoreductase	90	13.09	1.07E–13
Mitochondrion	136	11.76	3.73E–12
Signal peptide/secreted	107	5.19	5.52E–07
Respiratory chain	23	4.04	4.86E–04
Amino acid metabolism	15	3.97	2.53E–05
Glutathione metabolism	16	3.13	4.17E–05
Fatty acid/lipid metabolism	24	3.03	9.26E–04
Aldehyde dehydrogenase	8	2.67	7.41E–04
Calcium	42	2.30	3.21E–05
Cytochrome P450	16	2.16	6.21E–04
Protease inhibitor	11	2.14	4.81E–04

(d) Differentiated v undifferentiated down-regulated			
Annotation cluster	Count	Enrichment score	*p* value
Ribosomal protein	86	38.95	4.90E–38
Protein biosynthesis	61	9.24	1.39E–24
Chaperone	47	7.73	5.43E–11
Proteasome	39	7.64	1.59E–24
Ribosomal biogenesis	23	5.89	5.51E–08
Aminoacyl-tRNA synthesis	20	2.91	1.37E–07
ATPase activity	24	2.79	1.74E–04
ATP binding	110	2.40	3.61E–04

(a) Functions up-regulated in differentiated organoids compared to liver. (b) Functions down-regulated in differentiated organoids compared to liver. (c) Functions up-regulated in differentiated compared to undifferentiated organoids. (d) Functions down-regulated in differentiated compared to undifferentiated organoids

Table 1b shows that the proteins exhibiting decreased expression in differentiated organoids compared to liver were typically present at increased levels in differentiated compared to undifferentiated organoids (Table 1c). For example, proteins involved in metabolism of xenobiotics by CYP450 and those involved in fatty acid, glutathione and amino acid metabolism were reduced in differentiated organoids compared to liver *but* increased in differentiated versus undifferentiated organoids (Table 1c). This suggests that, in terms of metabolism, differentiated organoids are closer to the liver phenotype than their undifferentiated counterparts. Proteins that were down-regulated between differentiated and undifferentiated organoids included those involved in RNA translation and protein synthesis/turnover (Table 1d).

### IPA reveals critical deficiencies in the organoid proteome

**Fig. 4:**
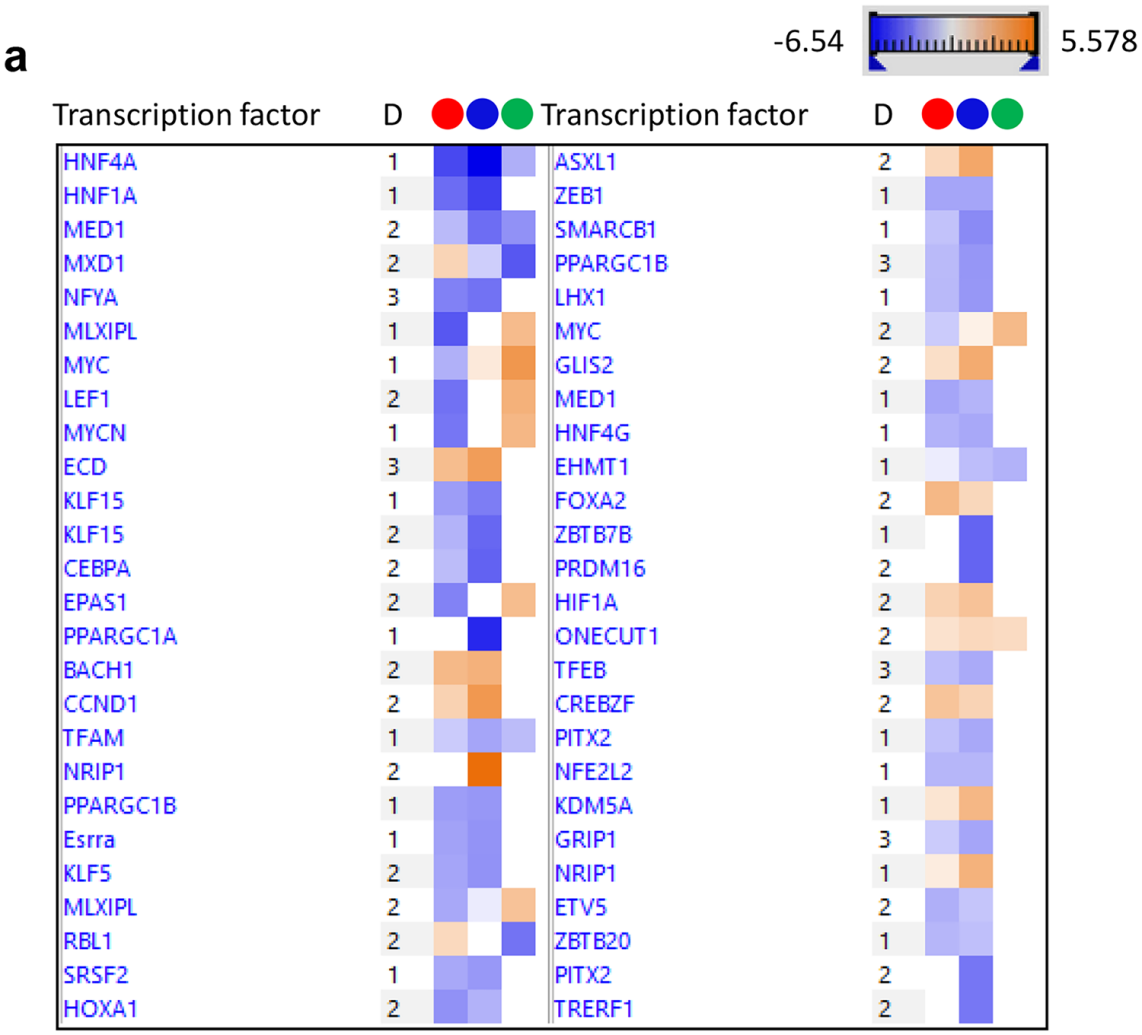

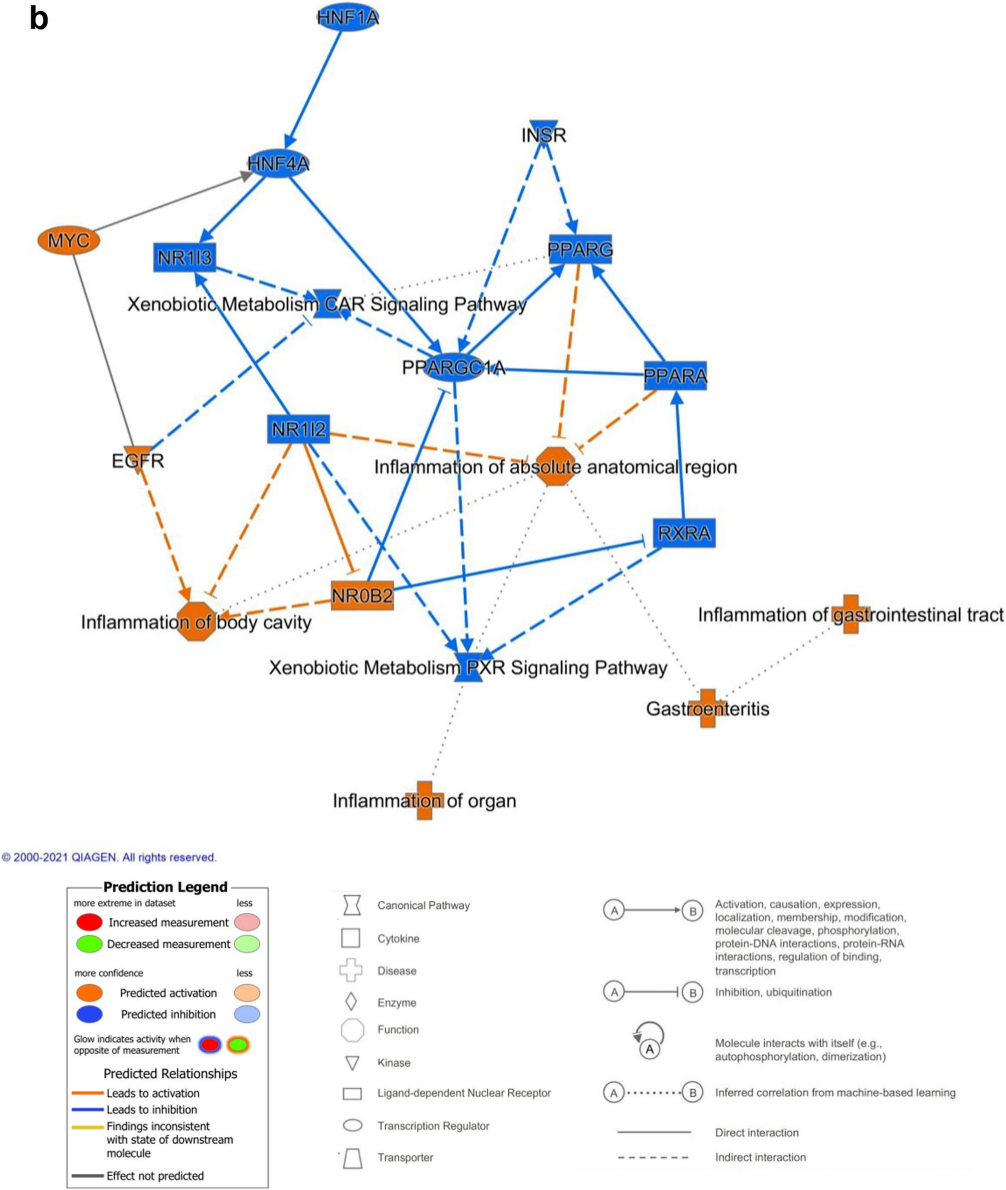
Ingenuity pathway analysis of proteins differentially expressed between undifferentiated organoids, differentiated organoids and liver. **a** Heatmap of the transcription factors predicted to be responsible for the observed phenotypes.  differentiated v liver,  undifferentiated v liver,  differentiated v undifferentiated. **b** Graphical summary of the relative activity of key proteins in undifferentiated compared to differentiated organoids (color figure online)

Analysis of diseases and functions within IPA revealed that organoids, whether differentiated or not, exhibited increased expression of proteins associated with organismal injury and abnormalities compared to liver (Supplementary Figure 3). As already illustrated in Fig. 3, proteins associated with metabolic functions were present at lower levels in the organoids, with differentiation alleviating this slightly. Canonical pathway analysis suggested that signaling via the retinoid X receptor (RXR), the farnesoid X receptor (FXR) and xenobiotic receptor (PXR) was lower in the organoids (data not shown). These receptors coordinate the transcription of genes involved in xenobiotic metabolism and inflammation. Finally, upstream analysis showed that key transcription factors were predicted to be responsible for the differences between the liver and the organoid phenotype (Fig. 4a). These included hepatocyte nuclear factor 4-alpha (HNF4A), HNF1A, carbohydrate-responsive element-binding protein (MLXIPL), mediator of RNA polymerase II transcription subunit 1 (MED1), Myc proto-oncogene (MYC) and MAX dimerization protein 1 (MXD1). Differentiation of organoids did not result in a marked difference in the proteome, but a nuanced alteration in the protein expression profile was observed. This is illustrated in the graphical summary in Fig. 4b, where the expression of the key transcription factors HNF4A and HNF1A were predicted to be lower in undifferentiated organoids compared to their differentiated counterparts.

### Validation of mass spectrometry data by immunoblotting

To validate the differential expression of proteins detected by iTRAQ, the expression of a CYP450 (3a11), phase II (GSTA1), DILI biomarker (ALT1) and the NRF2-associated protein peroxiredoxin 1 (PRDX1) were further analyzed by immunoblotting (*n* = 2 per group). Samples from day four and day eight of differentiation were also included to show the change of protein induction over time.

**Fig. 5:**
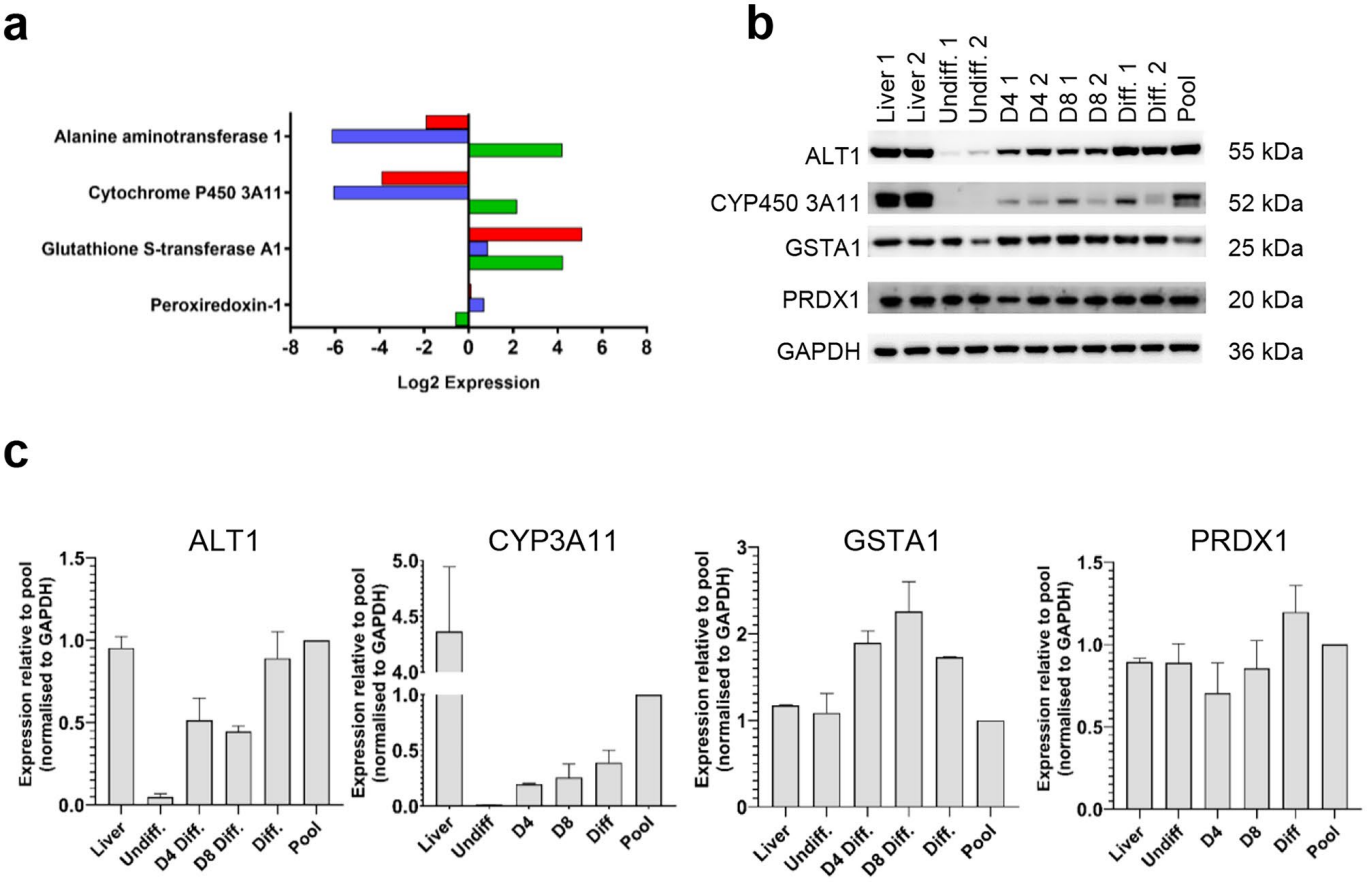
Comparison of relative protein expression levels by iTRAQ and immunoblotting. **a** Log2 fold difference in expression of DMET and NRF2 proteins detected by iTRAQ. **b** Immunoblot of DMET and NRF2 proteins in liver and hepatic organoids at various stages of differentiation (*n* = 2). **c** Densitometric analysis of DMET and NRF2 proteins relative to GAPDH showed a similar protein expression to that observed by iTRAQ analysis.  differentiated v liver,  undifferentiated v liver,  differentiated v undifferentiated (color figure online)

The result of the iTRAQ abundance data (Fig. 5a) was compared to immunoblot (Fig. 5b) for the same markers. The trend in protein expression by immunoblot reflected iTRAQ data. Densitometry analysis was performed to quantitatively assess changes in protein expression (Fig. 5c). Band intensities calculated relative to GAPDH demonstrated an increase in CYP3a11, GSTA1 and ALT1 over time. The amount of PRDX1 remained constant throughout differentiation. Overall, changes in protein expression determined by immunoblotting were shown to be an accurate reflection of the data obtained from the iTRAQ analysis.

### Investigation of the effect of differentiation on the response of the differentiated organoids to dose-dependent toxicity

**Fig. 6:**
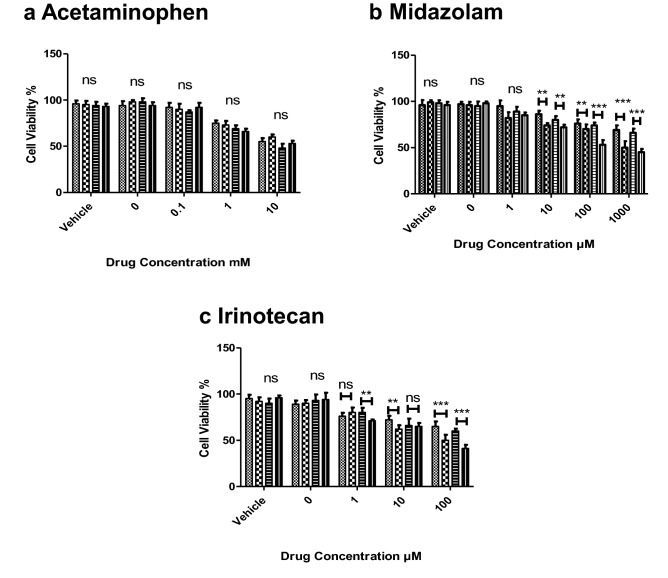
Cell viability (LDH) in response to pharmaceutical compounds. **a** LDH cell viability in response to APAP (0,0.1,10,10 mM) over 24 and 48 h (*n* = 3). **b** LDH cell viability in response to midazolam (0, 1, 10, 100, and 1000 µM) over 24 and 48 h (*n* = 3). **c** LDH cell viability in response to irinotecan (0, 1, 10, and 100 µM) over 24 and 48 h (*n* = 3). All doses/time points were replicated in both differentiated and undifferentiated states. One-Way ANOVA was performed on differentiated vs undifferentiated on parallel dose/time points. Ns = not significant, * = 0.1, ** = 0.05, *** = 0.01.  24 h undifferentiated,  24 h differentiated,  48 h undifferentiated,  48 h differentiated (color figure online)

APAP induced a time- and dose-dependent decrease in cell viability, decreasing from 96% ± 3.2 when untreated, to ~ 55% ± 7.0 when treated with 10 mM APAP. Organoids did not display a significant difference in susceptibility to APAP toxicity when differentiated towards a hepatocyte-like fate (Fig. 6a).

Midazolam also induced a time- and dose-dependent decrease in cell viability. However, in this case differentiation resulted in increased susceptibility to midazolam-induced toxicity, with cell viability decreasing from 66% ± 5.1 in undifferentiated organoids to 45% ± 3.5 in differentiated organoids when dosed with 1000 µM midazolam for 48 h (Fig. 6b).

Finally, irinotecan induced a time- and dose-dependent decrease in cell viability. Cell viability decreased from 60% ± 2.5 in undifferentiated organoids to 41% ± 4.1 in differentiated organoids following treatment with 100 µM Irinotecan for 48 h (Fig. 6c).

## Discussion

The value of any cultured cell as a disease model lies in its ability to robustly reflect the in vivo phenotype. While primary hepatocytes are the current gold standard for modeling the liver, their availability, reproducibility and DMET stability in culture negatively affect their widespread use. Hepatic organoids are a novel, but rather poorly characterized, in vitro system that may meet the need for a more physiologically relevant, proliferative, liver-derived cell model. In this study, the global proteomes of undifferentiated and differentiated murine organoids and donor-derived liver tissue were compared to better characterize this nascent hepatic model.

The in vitro proliferation of hepatocytes is typically not possible under standard culture conditions, and therefore, requires extensive measures such as viral transfection (Levy et al. 2015) or culture with tumor necrosis factor α (Peng et al. 2018). Conversely, it was observed that hepatic organoids from a single donor could be expanded and passaged multiple times in basal expansion media. Biliary-derived organoids cultured with Notch signaling inhibition and dexamethasone treatment exhibit a mature hepatocyte phenotype (Broutier et al. 2016). Here, at the final stage of differentiation, organoids expressed low levels of AFP, and a subset of organoids expressed albumin; however, they remained CK19^+^ (Supplementary Figure 1). This suggests that differentiation of organoids does not induce a complete trans-differentiation from a biliary to hepatocyte phenotype, only a gain of hepatocyte function. Li et al. (2014) demonstrated that human hepatic progenitor cells cultured in hepatocyte medium were CK19^+^/AFP^+^/Albumin^+^, while fully differentiated human organoids have also been shown to be fpCAM^+^/HNF4α^+^/Albumin^+^ and CK19^+^/Albumin^+^/CYP3A4^+^ (Huch et al. 2015). This shift toward a hepatocyte-like cell was confirmed by functional annotation cluster analysis, with the upregulation of multiple metabolic and CYP450 pathways in differentiated organoids compared to undifferentiated organoids (Table 1 and Fig. 3). Further evidence for phenotypic change is reflected by the tighter clustering of undifferentiated and differentiated organoids compared to liver tissue by hierarchical and PCA (Supplementary Figures 2a and b). The same tight clustering of organoids relative to donor-matched liver tissue has also been observed on a gene level by whole-genome transcriptomic analysis of essential liver function markers (Huch et al. 2015).

The expression of DMET proteins in hepatic organoids has not been comprehensively defined prior to this study. Within our dataset, the differentiation protocol partially restored the expression of the majority of CYP450 enzymes, though not to the level observed in the liver. The murine equivalent families of CYP3a4, 2d6, 2c19, 1a2 and 2b6, which oxidatively metabolize the most commonly prescribed drugs (Zanger et al. 2008), were all significantly up-regulated in differentiated organoids compared to undifferentiated organoids (Fig. 3a). Organoids dosed with acetaminophen exhibited dose-dependent toxicity (Fig. 6a), suggesting that active Cyp2e1 was expressed. However, there was no significant change in CYP2e1 expression upon differentiation (Fig. 3a), suggesting that only a basal level of expression was achieved. Liver spheroids and liver-on-a-chip models showed similar toxicity profiles when exposed to APAP at 0–10,000 µM (Foster et al. 2019). It is also possible that metabolism-independent toxicity due to APAP exposure underlies the dose-dependent toxicity that we saw in both the differentiated and undifferentiated organoids. Metabolism inhibition using, for example, 1-aminobenzotriazole will be necessary to confirm this. Previous work has shown that differentiated human hepatic organoids demonstrated CYP3A4 activity and were capable of metabolizing midazolam, a commonly used probe of CYP3A4 activity (Arellano et al. 2007; Majumdar et al. 2003). In agreement with these studies, the expression of CYP3a proteins in our data increased in differentiated murine hepatic organoids following culture with dexamethasone (Down et al. 2007; Matoulková et al. 2014) and cell viability decreased following treatment with midazolam and irinotecan (Fig. 6b, c). The metabolism of midazolam is not strictly unique to CYP3A4 as this drug is also turned over by CYP450 3A3 and 3A5 (Wandel et al. 1994). Interestingly, the level of CYP3a13 (equivalent to human CYP3A5) was the only CYP450 enzyme significantly higher in differentiated organoids compared to both liver and undifferentiated organoids and this may be primarily responsible for the midazolam metabolism seen in the original study (Huch et al. 2015). Midazolam hepatotoxicity has not been widely reported in the literature. However, it has been shown to decrease proliferation and increase apoptosis in HepG2 cells (Qi et al. 2020). It is possible that the low levels of UGTs in differentiated organoids in conjunction with the relative high levels of Cyp3a11 and 3a13 make them more susceptible to the metabolite of midazolam than would be the case in fully metabolically competent hepatocytes (Hyland et al. 2009).

Finally, differentiation induced high expression of CYP2c55 and 2s1 (Fig. 3a). While relatively little is known about these CYP450 enzymes, van Waterschoot et al. (2007) observed a > 30-fold increase in gene expression of CYP2c55 in a CYP3a^−^/^−^ mouse model, which was capable of midazolam metabolism. CYP2s1 has been shown to be mainly expressed in extrahepatic tissue within epithelial cells and to metabolize retinoic acid, a key driver of differentiation (Saarikoski et al. 2005). The increase of these two CYP450 proteins in fully differentiated hepatic organoids, therefore, warrants further investigation.

At the time of writing, this is the first study to profile phase II and hepatic transporters in biliary-derived hepatic organoids. Differentiation up-regulated the expression of the GST and sulfotransferase family, with many proteins expressed at near-equivalent (GST-M2, sulfotransferase 1A1) or higher (GSTA1, sulfotransferase 1C2) levels compared to the liver (Fig. 3b). Although hepatic transporters generally had lower expression in organoids compared to liver, the magnitude of difference was relatively small compared to CYP450 and phase II proteins. There are a number of essential hepatobiliary influx and efflux transporters expressed in differentiated organoids, such as MDR1A, MRP1/2/3/6/7, OCT1, OATP-1, BSEP, BCRP and the sodium/bile acid cotransporter (Fig. 3c). Critically, many of these proteins are lacking in currently used hepatic models, such as HepG2 (Poloznikov et al. 2018; Wilkening et al. 2003). These properties are likely to make the organoids useful for specific absorption, distribution, metabolism and excretion (ADME) studies, relative to pre-existing models, perhaps as a component of a Tier 2 hepatotoxicity safety assessment strategy, as we set out recently (Weaver et al. 2020).

IPA analysis revealed the potential role of critical transcription factors in the establishment of the hepatic phenotype (Fig. 4a), and provides some insight into the limitations of the hepatic organoid differentiation protocol. HNF4A has recently been identified as a critical transcription factor in the differentiation of endodermal cells to hepatic progenitors (DeLaForest et al. 2018). It does this by recruiting RNA polymerase II to a pre-initiation complex with Mediator and other transcription factors. The reduced expression of HNF4A, MED1 and MYC proteins and the elevated expression of MXD1 (antagonizes Myc transcriptional activity) in organoids compared to liver all point to a dysregulation of RNA polymerase II mediated transcription of protein encoding genes. Whilst the level of HNF4A is predicted to be elevated in differentiated compared to undifferentiated organoids (Fig. 4b), further increasing the level of this transcription factor may help to achieve a more hepatocyte-like phenotype (Cheng et al. 2019; Song et al. 2016). IPA also reveals gene expression in relation to specific disease states, i.e., inflammation, cirrhosis or cancer (Supplementary Figure 3), and this highlights the possible modeling of disease states with our organoid model.

To some extent, parallels can be drawn between hepatic organoids and HepaRG cells as they are both derived from a progenitor cell that requires differentiation to a hepatobiliary phenotype, and both exhibit some CYP3A expression (Marion et al. 2010). Unlike HepaRG, hepatic organoids are not cancer-cell derived; thus, they may have a more favorable NRF2 and bioenergetic profile for investigating hepatocellular toxicity. The levels of free radical production and NRF2 activation in HepaRG varies throughout differentiation, which may impair the mechanistic understanding of drug-induced cellular defense activation (Bellanti et al. 2017). Within this dataset, no significant difference in the levels of KEAP1, heme oxygenase-1 or NAD(P)H dehydrogenase [quinone] 1 between liver and hepatic organoids was detected, indicating a stable NRF2 system throughout the organoid culture (Fig. 3d). It is possible that the use of 2D culture for HepaRG and 3D culture for hepatic organoid may account for some of these differences. Nevertheless, differentiated organoids expressed the most commonly used current DILI biomarkers at a similar level to the liver, which would aid in their use as a translational tool for evaluating hepatotoxicity (Fig. 3e).

Hepatic organoids may offer utility compared to other advanced hepatic models such as HepaRG, spheroid and induced pluripotent stem cell models. Unlike HepaRG cells, hepatic organoids form a non-cancerous, patient-specific donor cell and reach differentiation in a shorter timeframe. While spheroids and simple 2D cultures can be formed from patient-specific hepatocytes, their lack of availability, proliferation and de-differentiation in culture limits their use in routine drug testing. Finally, though iPSC cells are non-cancerous and patient-specific, the lack of a standardized differentiation protocol, rather immature phenotype and technically challenging 3D culture currently restricts their utility in DILI assessment.

We have shown that differentiated hepatic organoids express high levels of certain essential metabolizing and transport proteins, such as CYP3A, GSTA and MDR1A. Therefore, hepatic organoids may be the most appropriate model for assessing drug classes that are processed through these pathways, such as such as alkylating chemotherapy agents (Ekhart et al. 2009). Furthermore, the expression of multiple hepatobiliary transporters may enable mechanistic insight into biliary efflux of metabolized compounds and inform on potential cholestatic perturbations, which are typically difficult to model in vitro (Vinken 2018).

For in vitro toxicological studies, we will need to be careful with respect to the accuracy and normalization of seeding organoid fragments compared to an exact cell count seen in the other hepatic models. Further investigation to determine which model is fit for a specific purpose is, therefore, required. Since the original publication of biliary-derived organoids (Huch et al. 2015), a recent paper has described organoid outgrowth from albumin^+^/Axin2^+^ hepatocytes. Compared to biliary-derived organoids, gene expression of cholangiocyte/hepatic progenitor cell markers (CK19, SOX9) was significantly lower and hepatic markers (albumin, hepatocyte nuclear factor 4α and CYP1a2 and 3a11), were comparable to primary hepatocytes, which may offer a more physiologically relevant hepatocyte model (Hu et al. 2018). As the field of hepatic organoids is relatively novel, organoid isolation and differentiation protocols will require further experimentation, optimization and characterization.

The current study has shown that hepatic organoids have the potential to improve and/or replace current in vitro models for toxicity and safety testing of xenobiotics. We consider that the use of proteomics to track the differentiation protocol is a powerful one, it will next be applied to human-derived hepatic organoids, and further we believe that this approach should be considered for assessing the similarity to native tissue of organoids derived from other tissue types. Assessing the differences and similarities between in vitro dosing of hepatic organoids and in vivo toxicological studies would aid the understanding of how human-derived organoids could predict clinically observed toxicity. Ultimately, this could identify translational differences which may help predict complexities when moving from pre-clinical models to phase I trials. Furthermore, hepatic organoids should be challenged with curated hepatotoxic test and training compounds to evaluate their response to toxicological perturbation, with both standardized cytotoxic and mechanistic end-points. This response should then be contextualized relative to existing 2D and 3D in vitro models to assess their utility as a predictive and sensitive model of DILI.

## Supplementary Information

Below is the link to the electronic supplementary material.

Supplementary file1 (PPTX 979 kb)

## Data Availability

The mass spectrometry proteomics data have been deposited to the ProteomeXchange Consortium via the PRIDE partner repository with the dataset identifier PXD017986.
