# Supplementary material for: Proteomic profiling of murine biliary-derived hepatic organoids and their capacity for drug disposition, bioactivation and detoxification
Source: Arch Toxicol. 2021 May 29;95(7):2413–30. doi: 10.1007/s00204-021-03075-3 (PMC8241807; doi:10.1007/s00204-021-03075-3)
Supplement: Supplementary file 1 — Supplementary file1 (PPTX 979 kb) [file 204_2021_3075_MOESM1_ESM.pptx]

## Slide 1
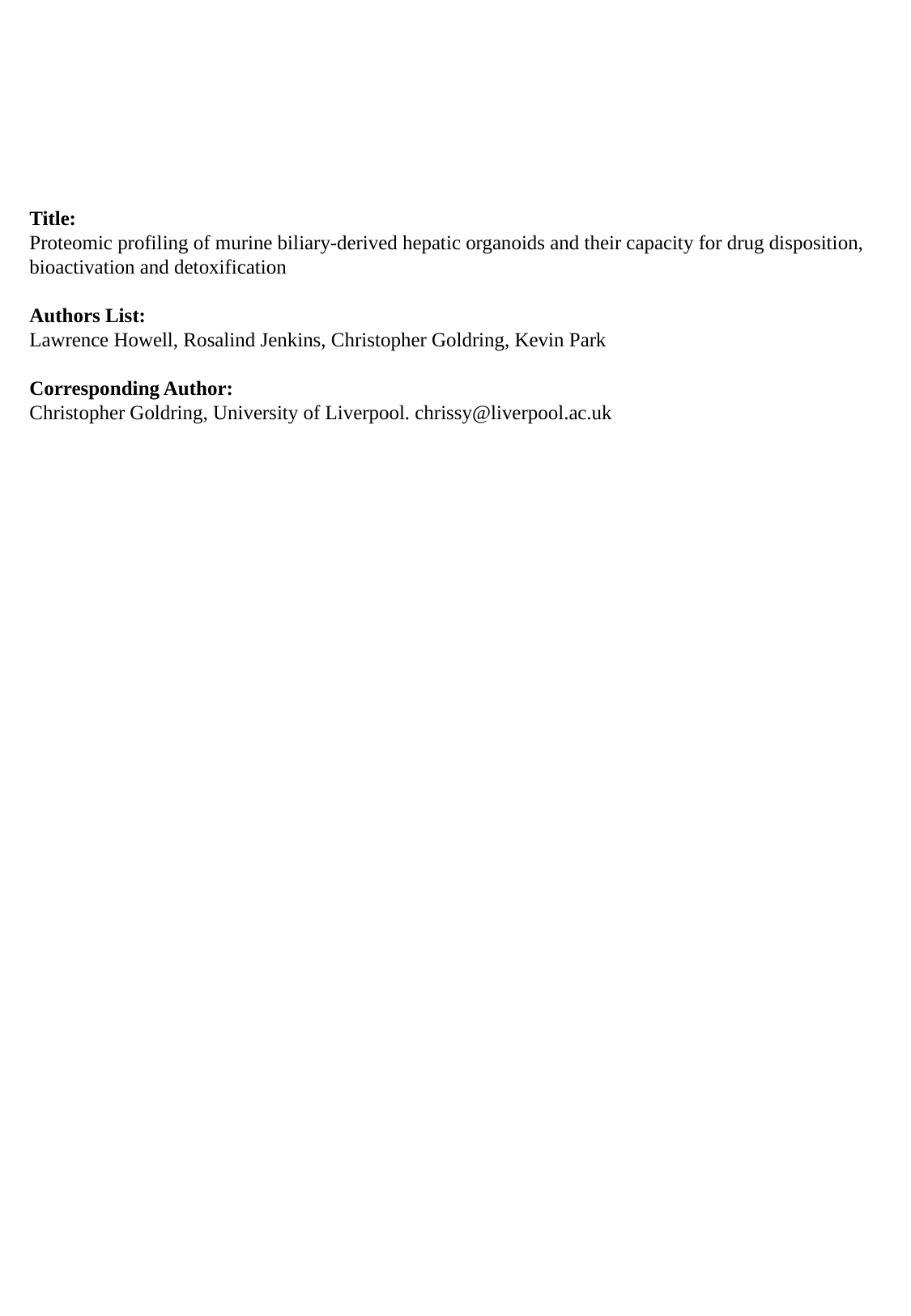

Title:
Proteomic profiling of murine biliary-derived hepatic organoids and their capacity for drug disposition, bioactivation and detoxification
Authors List:
Lawrence Howell, Rosalind Jenkins, Christopher Goldring, Kevin Park
Corresponding Author:
Christopher Goldring, University of Liverpool. chrissy@liverpool.ac.uk

## Slide 2
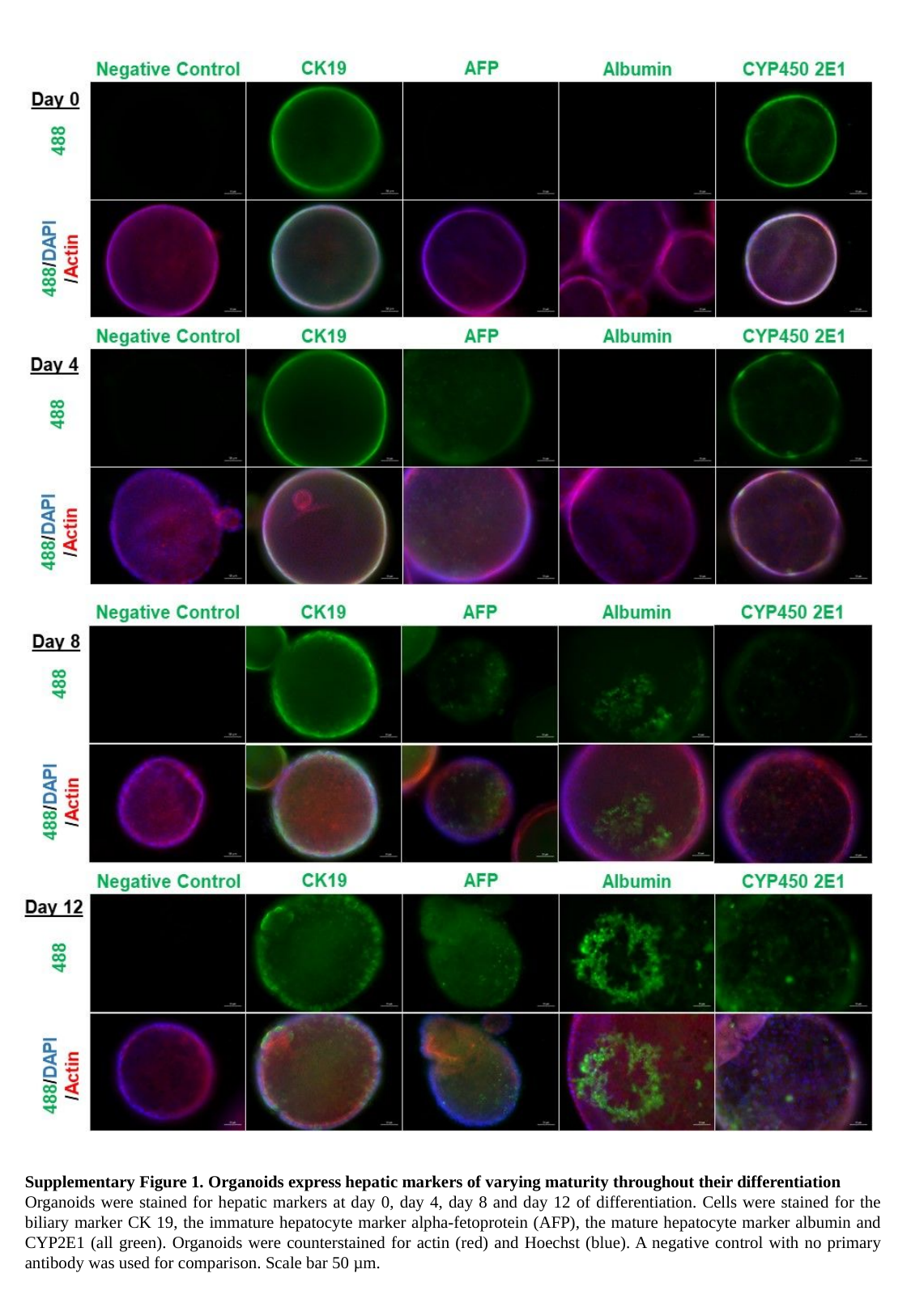

Supplementary Figure 1. Organoids express hepatic markers of varying maturity throughout their differentiation
Organoids were stained for hepatic markers at day 0, day 4, day 8 and day 12 of differentiation. Cells were stained for the biliary marker CK 19, the immature hepatocyte marker alpha-fetoprotein (AFP), the mature hepatocyte marker albumin and CYP2E1 (all green). Organoids were counterstained for actin (red) and Hoechst (blue). A negative control with no primary antibody was used for comparison. Scale bar 50 µm.

## Slide 3
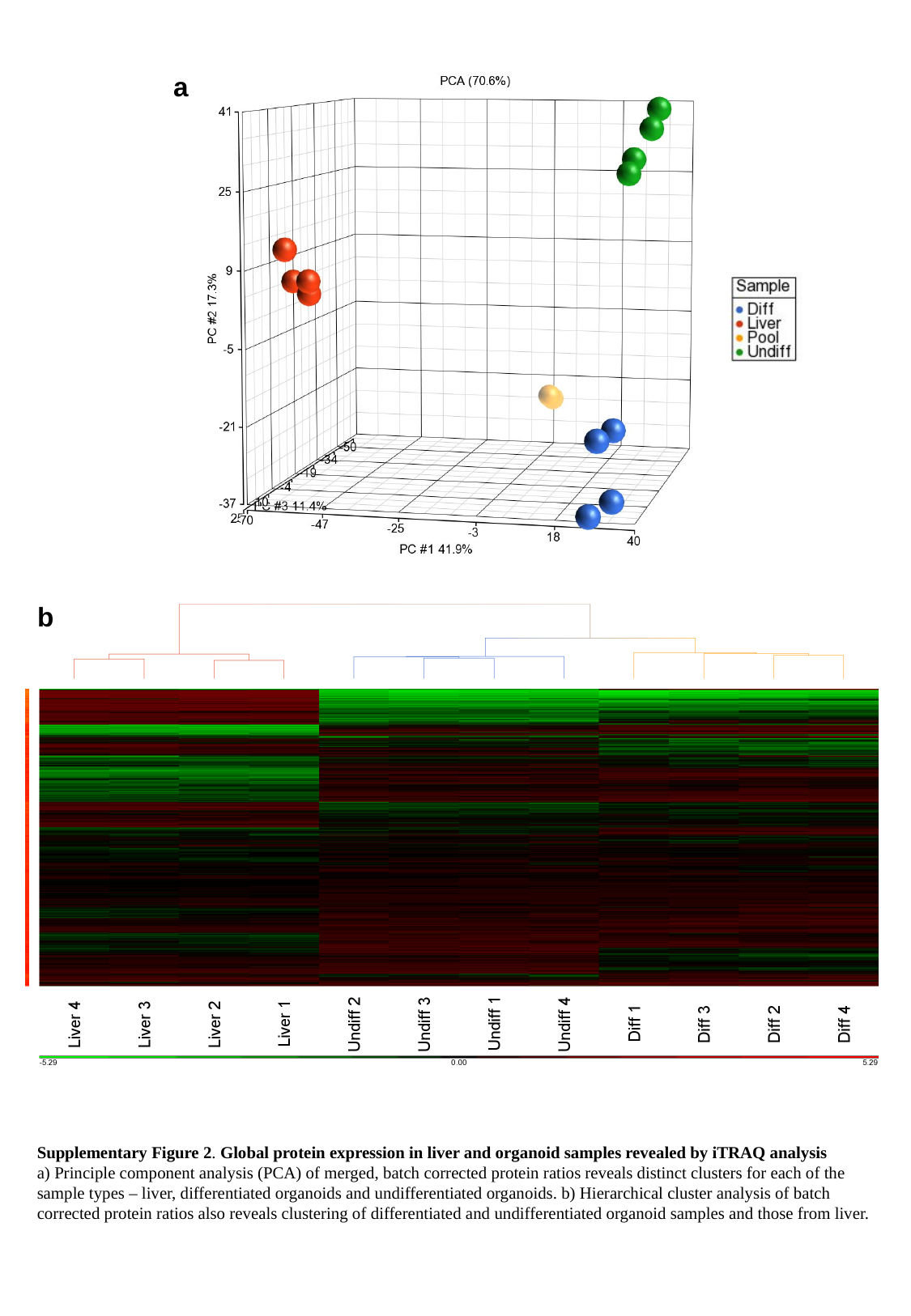

a
b
Supplementary Figure 2. Global protein expression in liver and organoid samples revealed by iTRAQ analysis
a) Principle component analysis (PCA) of merged, batch corrected protein ratios reveals distinct clusters for each of the sample types – liver, differentiated organoids and undifferentiated organoids. b) Hierarchical cluster analysis of batch corrected protein ratios also reveals clustering of differentiated and undifferentiated organoid samples and those from liver.

## Slide 4
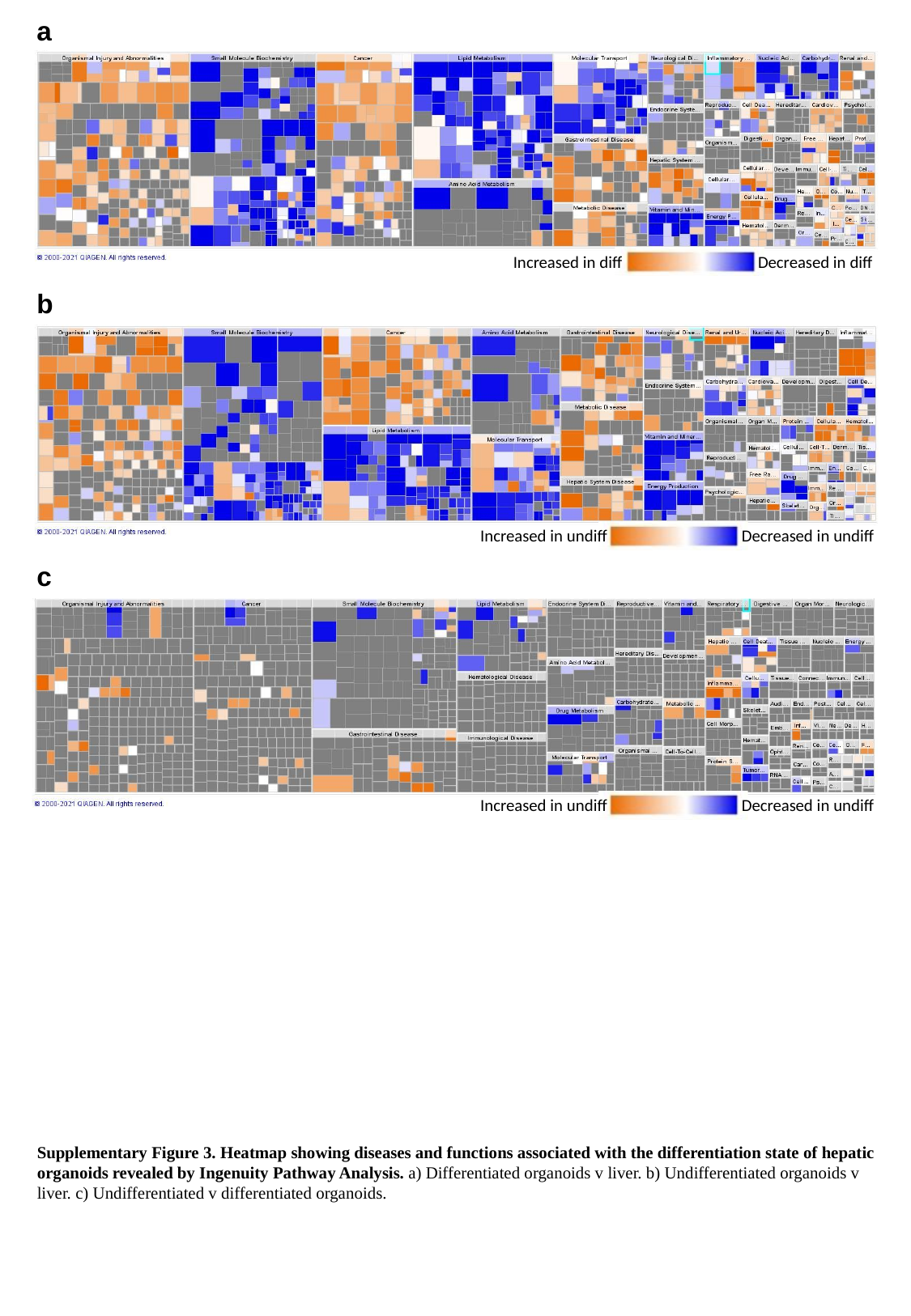

a
Increased in diff
Decreased in diff
b
Increased in undiff
Decreased in undiff
c
Increased in undiff
Decreased in undiff
Supplementary Figure 3. Heatmap showing diseases and functions associated with the differentiation state of hepatic organoids revealed by Ingenuity Pathway Analysis. a) Differentiated organoids v liver. b) Undifferentiated organoids v liver. c) Undifferentiated v differentiated organoids.
